# Multi-omics Characterization of Response to PD-1 Inhibitors in Advanced Melanoma

**DOI:** 10.3390/cancers15174407

**Published:** 2023-09-03

**Authors:** Lucía Trilla-Fuertes, Angelo Gámez-Pozo, Guillermo Prado-Vázquez, Rocío López-Vacas, Virtudes Soriano, Fernando Garicano, M. José Lecumberri, María Rodríguez de la Borbolla, Margarita Majem, Elisabeth Pérez-Ruiz, María González-Cao, Juana Oramas, Alejandra Magdaleno, Joaquín Fra, Alfonso Martín-Carnicero, Mónica Corral, Teresa Puértolas, Ricardo Ramos-Ruiz, Antje Dittmann, Paolo Nanni, Juan Ángel Fresno Vara, Enrique Espinosa

**Affiliations:** 1 Molecular Oncology Laboratory, Hospital Universitario La Paz-IdiPAZ, 28046 Madrid, Spain; lucia.lt30@gmail.com (L.T.-F.); angelogamez@gmail.com (A.G.-P.); 14gprado@gmail.com (G.P.-V.); rlvacas@gmail.com (R.L.-V.); juanangel.fresno@salud.madrid.org (J.Á.F.V.); 2 Biomedica Molecular Medicine SL, 28049 Madrid, Spain; 3 Instituto Valenciano de Oncología, 46009 Valencia, Spain; vsoriano-fivo@hotmail.es; 4 Spanish Melanoma Group (GEM), 08024 Barcelona, Spain; fernando.garicanogoldaraz@osakidetza.eus (F.G.); mjlecumberri@yahoo.es (M.J.L.); mararch3@hotmail.com (M.R.d.l.B.); mmajem@santpau.cat (M.M.); eliperu@gmail.com (E.P.-R.); mgocao@gmail.com (M.G.-C.); juanaoramas@gmail.com (J.O.); magdaleno.cremades@gmail.com (A.M.); jfrar@seom.org (J.F.); monicacorral72@hotmail.com (M.C.); tjpuertolas@gmail.com (T.P.); 5 Hospital de Galdakao, 48960 Galdakao, Spain; 6 Complejo Hospitalario de Navarra, 31008 Pamplona, Spain; 7 Hospital de Valme, 41014 Sevilla, Spain; 8 Hospital de la Santa Creu i Sant Pau, 08001 Barcelona, Spain; 9 Unidad de Gestión Clínica Intercentros (UGCI) de Oncología Médica, Hospitales Universitarios Regional y Virgen de la Victoria, Instituto de Investigación Biomédica de Málaga (IBIMA), Hospitales Universitarios Regional y Virgen de la Victoria, 29010 Málaga, Spain; 10 Hospital Quirón Dexeus, 08028 Barcelona, Spain; 11 Hospital Universitario de Canarias-San Cristóbal de la Laguna, 38320 Tenerife, Spain; 12 Hospital Universitario de Elche y Vega Baja, 03203 Alicante, Spain; 13 Hospital Universitario Río Hortega, 47012 Valladolid, Spain; 14 Hospital San Pedro, 27347 Logroño, Spain; 15 Hospital Clínico Lozano Blesa, 50009 Zaragoza, Spain; 16 Hospital Universitario Miguel Servet, 50009 Zaragoza, Spain; 17 Genomics Unit, Parque Científico de Madrid, 28049 Madrid, Spain; ricardo.ramos@fpcm.es; 18 Functional Genomics Center Zurich, University/ETH Zurich, 8092 Zurich, Switzerland; antje.dittmann@fgcz.ethz.ch (A.D.); paolo.nanni@fgcz.uzh.ch (P.N.); 19 CIBERONC, ISCIII, 28222 Madrid, Spain; 20 Medical Oncology Service, Hospital Universitario La Paz, 28046 Madrid, Spain

**Keywords:** melanoma, immunotherapy response, multi-omics, inflammatory response, protein processing in the endoplasmic reticulum

## Abstract

**Simple Summary:**

The survival of advanced melanoma patients has been improved in recent years due to immunotherapy. However, not all patients respond to this treatment. For this reason, it is necessary to know the mechanisms of the response and resistance to immunotherapy. In this work, clinical samples from advanced melanoma patients treated with immunotherapy were analyzed. The obtained results suggested that the proteins involved in protein processing in the endoplasmic reticulum and antigen presentation, as well as the immune and inflammatory responses, play a role in the response to immunotherapy. Additionally, we built a prognostic signature capable of identifying those patients that will respond to immunotherapy. The study of the mechanisms of the resistance and response to immunotherapy could help in the definition of new therapies for these patients that do not respond to immunotherapy.

**Abstract:**

Immunotherapy improves the survival of patients with advanced melanoma, 40% of whom become long-term responders. However, not all patients respond to immunotherapy. Further knowledge of the processes involved in the response and resistance to immunotherapy is still needed. In this study, clinical paraffin samples from fifty-two advanced melanoma patients treated with anti-PD-1 inhibitors were assessed via high-throughput proteomics and RNA-seq. The obtained proteomics and transcriptomics data were analyzed using multi-omics network analyses based on probabilistic graphical models to identify those biological processes involved in the response to immunotherapy. Additionally, proteins related to overall survival were studied. The activity of the node formed by the proteins involved in protein processing in the endoplasmic reticulum and antigen presentation machinery was higher in responders compared to non-responders; the activity of the immune and inflammatory response node was also higher in those with complete or partial responses. A predictor for overall survival based on two proteins (AMBP and PDSM5) was defined. In summary, the response to anti-PD-1 therapy in advanced melanoma is related to protein processing in the endoplasmic reticulum, and also to genes involved in the immune and inflammatory responses. Finally, a two-protein predictor can define survival in advanced disease. The molecular characterization of the mechanisms involved in the response and resistance to immunotherapy in melanoma leads the way to establishing therapeutic alternatives for patients who will not respond to this treatment.

## 1. Introduction

Melanoma is the most lethal cutaneous cancer [1,2]. In localized melanoma, resection is usually curative, but once the tumor spreads, the prognosis is poor. The prognosis of patients with advanced disease was dismal before the advent of targeted therapies and immunotherapy. The median of the overall survival of these patients was less than twelve months [3]. With the appearance of targeted therapies and immunotherapy, this outcome experienced an improvement. Now, the overall survival exceeds two years, and the five-year survival rate is 60% and 48% in BRAF-mutated and BRAF wild-type patients, respectively [1,2,4,5].

Targeted therapies are mainly based on the BRAF and MEK inhibitors, and they are employed in patients with mutations in the BRAF inhibitor [6,7]. Immunotherapy is mainly based on the PD-1 and CTLA-4 antibodies [8,9]. Immunotherapy improves overall survival, but less than 50% of patients become long-term survivors [1,8,10]. The response rates for anti-PD-1 monotherapy are approximately 40% and 60% for the combinations of anti-PD-1 and anti-CTLA-4 [11]. For this reason, it is important to gain insight into the mechanisms of resistance.

Genomics-based markers that predict responses to immunotherapy, such as PD-L1 expression, tumor mutational burden and microbiota, have been proposed, but are not used to make clinical decisions due to their poor accuracy [12,13]. The use of proteomics to reveal factors related to the response to immunotherapy in melanoma is very recent and may offer complementary information to genomics, being useful in the study of the direct effectors of biological processes. 

In this study, a molecular characterization of the response to anti-PD-1 inhibitors in advanced melanoma using transcriptomics and proteomics coupling with a Systems Biology analysis was performed, with the aim of defining the biological processes involved in response to anti-PD-1 inhibitors.

## 2. Materials and Methods

### 2.1. Spanish Melanoma Group Cohort

Fifty-two samples collected before treatment from patients with advanced melanoma were retrieved by the Spanish Melanoma Group (GEM). Patients had been treated with the anti-PD-1 inhibitors pembrolizumab or nivolumab. Approval from the Ethics Committee of the Comunidad Foral de Navarra (EO17/23) and written consent for each participant were obtained. The inclusion criteria were advanced melanoma, cutaneous or mucosal melanoma, and treatment with anti-PD-1 inhibitors (pembrolizumab or nivolumab), with formalin-fixed paraffin embedded (FFPE) samples and clinical information available. Exclusion criteria: uveal melanoma. Clinical responses were assessed radiologically as per standard clinical practice, and evaluated using the RECIST criteria.

Survival was measured from the initiation of anti-PD-1 therapy until death or last known follow-up. The Kaplan–Meier method was used to estimate survival.

### 2.2. Protein Isolation and Digestion

Protein isolation was carried out as previously described [14]. Briefly, FFPE sections were deparaffinized in xylene and washed twice in absolute ethanol. Protein isolates were diluted in 2% sodium dodecyl sulfate (SDS), and protein quantification was carried out using a MicroBCA Protein Assay Kit (Pierce, Thermo Fisher, Waltham, MA, USA). Ten µg of each protein isolate were digested with trypsin (1:50) and the SDS was removed using Detergent Removal Spin Columns (Pierce). Finally, samples were desalted using ZipTips (Millipore, Darmstadt, Germany), dried and resolubilized in 15 µL of a 0.1% formic acid and 3% acetonitrile solution.

### 2.3. Proteomics Experiments

Samples (5 µL of 1:10 dilution) were analyzed using a Q Exactive HF-X-Hybrid Quadrupole-Orbitrap mass spectrometer (Thermo Fischer Scientific, Bremen, Germany) coupled with M-class ultra-performance liquid chromatography (UPLC) (Waters). Peptide separation was performed using a commercial MZ Symmetry C18 Trap Column (100 Å, 5 µm, 180 µm × 20 mm, Waters) followed by a nanoEase MZ C18 HSS T3 Column (100 Å, 1.8 µm, 75 µm × 250 mm, Waters). The peptides were eluted at a flow rate of 300 nL/min at a gradient from 8 to 27% B in 85 min, 35% B in 5min and 80% B in 1 min. A mass spectrometer was operated in data-dependent acquisition mode (DDA), acquiring full-scan mass spectrometry (MS) spectra (350−100 *m/z*) at a resolution of 120,000 at 200 *m*/*z* after accumulation to a target value of 3,000,000, followed by HCD (higher-energy collision dissociation) fragmentation on the twenty most intense signals per cycle. HCD spectra were acquired at a resolution of 15,000 using a normalized collision energy of 25 and a maximum injection time of 22 ms. The automatic gain control (AGC) was set to 100,000 ions. Charge state screening was enabled. Singly, unassigned, and charge states higher than seven were rejected. Only those precursors with an intensity above 110,000 were selected for MS/MS. Precursor masses previously selected for MS/MS measurement were excluded from further selection for 30 s, and the exclusion window was set at 10 ppm. The samples were acquired using an internal lock mass calibration [15] on *m*/*z* 371.1012 and 445.1200. The MS proteomics results were handled using the local laboratory information management system (LIMS) [15]. The mass spectrometry proteomics data have been deposited to the ProteomeXchange Consortium via the PRIDE [16] partner repository with the dataset identifier PXD038155. Reviewers can access the data with the username reviewer_pxd038155@ebi.ac.uk and password Y8g4UyN1.

### 2.4. Proteomics Data Preprocessing

The acquired raw MS data were processed using MaxQuant version 1.6.2.3 [17], followed by protein identification using Andromeda [18]. Spectra were searched against the Uniprot reference proteome (taxonomy 9606, version 20190709), concatenated with its reverse decoy FastA database and common contaminants. Methionine oxidation and N-terminal protein acetylation were set as variable modifications. Enzyme specificity was set to trypsin/P allowing for a minimal peptide length of 7 amino acids and a maximum of two missed cleavages. The MaxQuant Orbitrap default search settings were used. The maximum false discovery rate (FDR) was set to 0.01 for peptides and 0.05 for proteins. Label-free quantification was enabled and a 2 min window for matching between runs was applied. In the MaxQuant experimental design template, each file was kept separate in the experimental design to obtain individual quantitative values.

Perseus software was used to transform protein data into log 2, apply quality criteria of at least two unique peptides and 50% of valid values, and impute missing values to a normal distribution using the default settings [19].

### 2.5. RNA Isolation

Five to ten 10–15 µm FFPE sections were obtained for each sample. The total RNA was isolated using a miRNeasy FFPE Kit (Qiagen, Venlo, The Netherlands) following the manufacturer’s instructions. The purified nucleic acid quality control for quantity and purity was assessed using an ND-1000 NanoDrop spectrophotometer (Thermo Fisher Scientific, Waltham, MA, USA).

### 2.6. RNA Capture and Sequencing

Relevant genes of melanoma related to the immune system, melanogenesis, keratinization and extracellular matrix were selected for this experiment, and 100 ng of RNA from each sample were used for library preparation with the KAPA RNA Hyperprep kit (Roche Nimblegen Inc., Madison, WI, USA) following the manufacturer’s instructions. A library fragment distribution was confirmed via electrophoresis and the concentration was determined using the KAPA library Quantification kit (Roche Nimblegen Inc.). A seven MB SeqCap EZ probe pool (Roche), including the genes previously defined, was designed using the NimbleDesign online tool. An equal mass of eight cDNA libraries was pooled and hybridized with the SeqCap EZ probe pool following the manufacturers’ specifications. The samples were sequenced in two groups using 2 × 100 pairs of the NextSeq 50/550 High Output Cartridge v2, 75 cycles. Mapping with TopHat and FPKM calculation using CuffLinks was performed using the G-Pro Suite [20]. The RNA-seq sequencing raw data files are available in Annotare (https://www.ebi.ac.uk/fg/annotare/ under the code accession E-MTAB-11729, accessed on 30 August 2023). 

### 2.7. Preprocessing of RNA Capture Data

First, the Ensembl gene notation was translated to the official gene symbols using the Ensembl Biomart release 100 tool (https://www.ensembl.org/biomart/, accessed on 30 August 2023) [21]. Seven gene symbols were duplicated, so the normalized counts of these genes were added to each other. Those genes with at least 400 counts in the 40 analyzed samples were selected. The data were log2 transformed and those genes with more than 50% of zeroes were removed. Finally, missing value imputation according to a normal distribution was performed using Perseus [19].

### 2.8. Multi-Omics Analysis Using Probabilistic Graphical Models

The proteomics and transcriptomics data were analyzed using a network analysis based on probabilistic graphical models (PGMs) to find a functional structure, as previously described [22,23]. The result of the PGM is an undirected graph with a local minimum Bayesian Information Criterion (BIC). This analysis was executed in two steps: first, the spanning tree with the maximum likelihood was built; second, a forward search that added edges and reduced the BIC, preserving the decomposability, was carried out [24]. The BIC penalizes the most complex models, obtaining the simplest possible graph of relations between the measured proteins and genes. PGMs have already demonstrated their utility in multi-omics analysis [23].

The PGM analysis was carried out using R 3.2.5 and the grapHD package [25]. Network visualization was carried out in Cytoscape 3.5software [26].

### 2.9. Search of Functional Structure

The obtained network in the PGM analysis was split into different branches in order to seek a functional structure. Gene ontology analyses of the proteins included in each branch were carried out using DAVID webtool v8 [27], using the GOTERM-FAT, Biocarta and KEGG categories and “Homo sapiens” as the background. The conversion between protein accession IDs and gene official symbols were carried out using Uniprot (https://uniprot.org/ (accessed on 30 August 2023)).

### 2.10. Functional Node Activity Calculation

With the aim of making comparisons in the activity of the different biological processes identified in the PGM network, functional node activities were calculated as the mean of the expression of those proteins/genes related to the main function of each functional node [23].

### 2.11. Statistical Analyses

To make comparisons between groups, the non-parametric U of the Mann–Whitney test was used. Statistical analyses were carried out using Graph Pad Prism v6. For survival analyses, the proteomics data were analyzed via Kaplan–Meier and log-rank testing, considering a *p*-value < 0.01 as statistically significant. Then, those proteins related to overall survival were used to build a predictive signature using Cox regression. These analyses were carried out using BRB Array Tools 4.6.2 [28].

## 3. Results

### 3.1. GEM Cohort

Fifty-two samples from patients diagnosed with advanced melanoma and treated with anti-PD-1 inhibitors (nivolumab or pembrolizumab) recruited by the Spanish Melanoma Group (GEM) were analyzed in this study.

**Figure 1:**
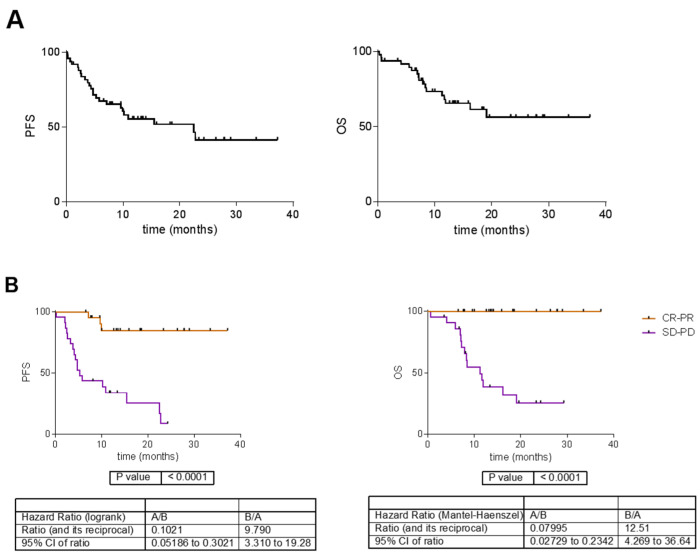
(**A**). Survival curves in the GEM cohort. (**B**). Survival curves according to best response. CR = complete response; PR = partial response; SD = stable disease; PD = progressive disease. PFS = progression-free survival; OS = overall survival.

Twenty-four out of the fifty-two patients had disease progression to PD-1 treatment, and eighteen died. Eleven patients had a complete response (CR), thirteen had a partial response (PR), ten had stable disease (SD) and thirteen had progressive disease (PD) as the best response. The remaining five patients were not evaluable for a response. The median of the progression-free survival (PFS) was 23 months and the median overall survival (OS) was not reached. As expected, responders (patients with complete response—CR— or partial response—PR) and non-responders (stable disease—SD—or progression—PD) had significant differences both in PFS and OS (Figure 1). All clinical characteristics of the patients are summarized in Table 1 and extended in Supplementary Table S1. For two patients, data on their OS were not available.

### 3.2. High-Throughput Proteomics Experiments

Fifty-two samples were analyzed via high-throughput proteomics. One sample was excluded because most of the protein present in the sample was hemoglobin. Three samples were excluded because of the quality of the MS2 measurements and five additional samples were excluded because they had more than 80% of the missing values across all the proteins. Finally, 43 of the 52 samples were used for the subsequent analyses.

A total of 5575 proteins were measured. After applying quality criteria (at least two unique peptides identified and 50% of valid values), 1225 proteins were used for the analyses.

### 3.3. RNA Capture Experiments

Fifty-two paraffin samples were retrieved, although four of them did not yield enough material to perform RNA extraction. After RNA extraction, eight samples were excluded due to a low RNA quantity yield. Therefore, 40 samples were analyzed via RNA-seq.

Of the analyzed genes, 2268 genes presented more than 400 lectures across the 40 patients and 2151 genes had less than 50% of zeroes.

In summary, the proteomics and transcriptomics information was available for 32 samples. Out of these 32 samples, 14 patients were responders, 13 were non-responders and 5 were non-evaluable.

### 3.4. Multi-Omics Systems Biology Analyses

**Figure 2:**
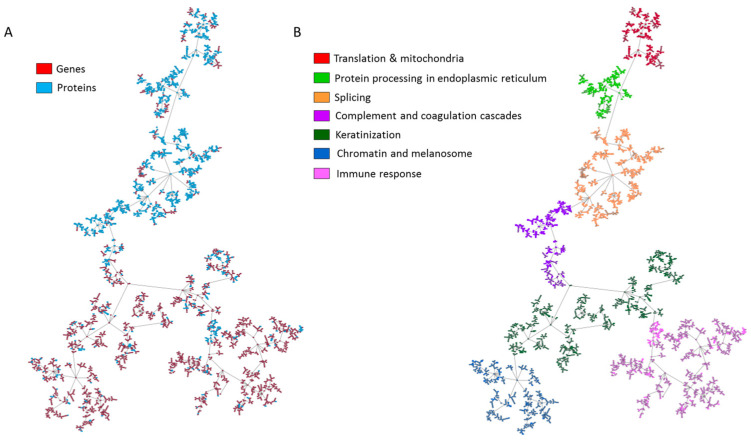
Network based on transcriptomics and proteomics experiments measured in advanced melanoma samples treated with anti-PD-1 inhibitors. (**A**) Location of genes and proteins in the network. (**B**) The network obtained using the probabilistic graphical model was split into seven functional nodes.

A probabilistic graphical model (PGM) using proteomics and transcriptomics data from 32 samples was built. The obtained network was split into seven functional nodes, two of them with two functions: chromatin and melanosome, and translation and mitochondria (Figure 2, Supplementary Table S2).

**Figure 3:**
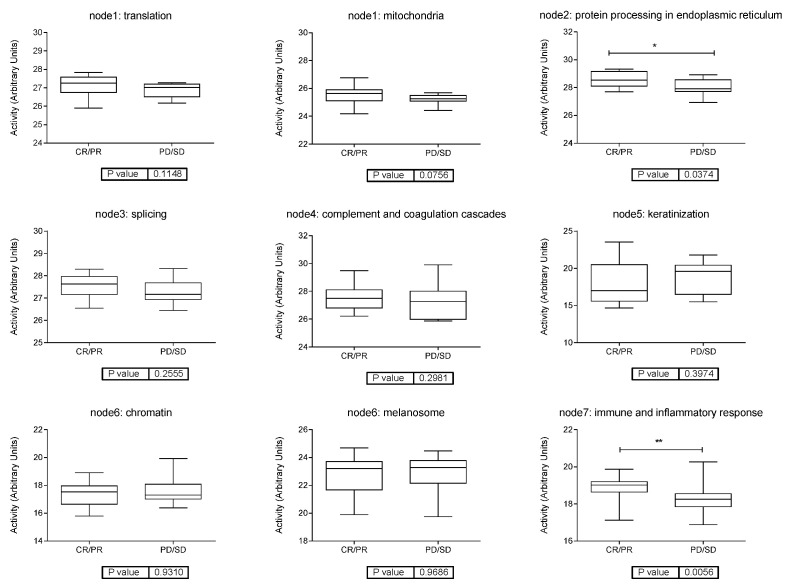
Functional node activity comparisons according to response to anti-PD-1 inhibitors. CR = complete response. PR = partial response. SD = stable disease. PD = progressive disease. * *p* < 0.05, ** *p* < 0.01.

Then, functional node activities were used to make comparisons between the groups. Regarding the response to anti-PD-1 inhibitors, we found differences in two functional node activities: “Protein processing in endoplasmic reticulum” and “immune and inflammatory response”. In both cases, the node activity was higher in tumors with CR and PR as the best responses compared to those with PD or SD (Figure 3). 

**Figure 4:**
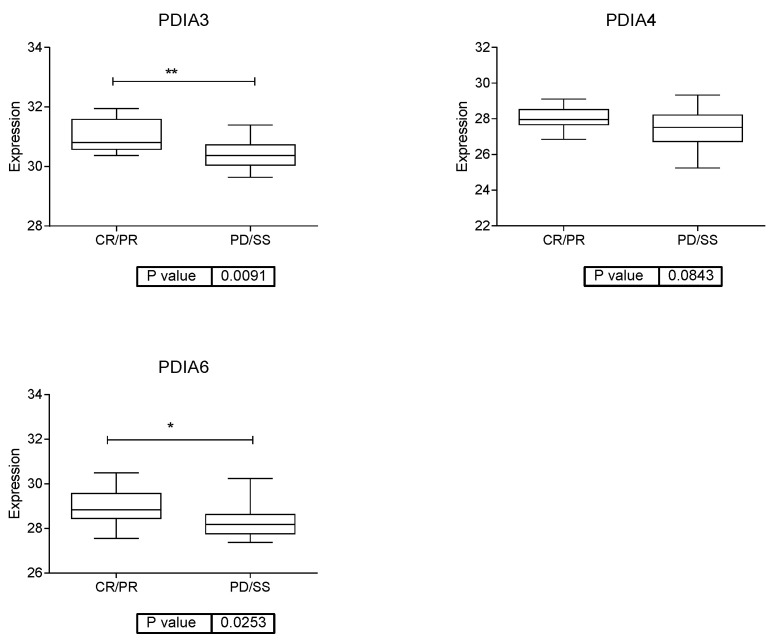
Expression of the main proteins in the node of protein processing in the endoplasmic reticulum. CR = complete response; PR = partial response; PD = progressive disease; SD = stable disease. * *p* < 0.05, ** *p* < 0.01.

The protein processing in the ER node contained proteins involved in folding proteins in the ER as PDIA3, PDIA4 and PDIA6, with a significantly higher expression in responders than in non-responders to anti-PD-1 therapy (Figure 4).

The immune and inflammatory response node contained 99 proteins and genes directly involved in the immune response such as interleukins and chemokines; T lymphocyte markers such as CD96, TLR8, CD80; or CCR5, beta-2-microglobulin (B2M) or absent in melanoma 2 (AIM2). Of these 99 proteins, 22 of them had a significantly differential expression between responders and non-responders, including Fas ligand, granzime, inducible T cell costimulator (ICOS), etc. (Supplementary Figure S1).

### 3.5. Proteomics Analyses

**Figure 5:**
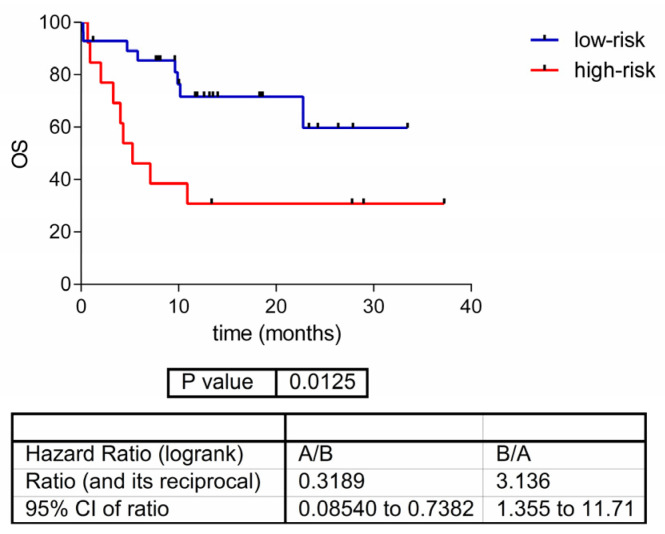
Overall survival predictor based on the expression of two proteins in the GEM cohort of patients with advanced melanoma treated with PD-1 inhibitors. OS = overall survival.

Using Kaplan–Meier and log-rank testing, two proteins were identified as being related to overall survival (*p* < 0.01): AMBP and PSMD5 (Table 2). Using these two proteins, an OS predictor was built using Cox regression (*p* = 0.012, HR = 3.13, 95% CI = (1.35–11.71)) (Figure 5). The low-risk group included 28 (68%) patients and the high-risk group contained 13 (32%) patients. The predictor was based on the following formula: 0.323 AMBP + 0.753 PSMD5 − 25.832. A sample was classified into the high-risk group if the prognosis index was higher than 0.409.

## 4. Discussion

An unmet need in the field of advanced melanoma is the prediction of the response to immunotherapy. In this study, the molecular landscape of melanoma samples treated with the anti-PD-1 inhibitors pembrolizumab and nivolumab was characterized using high-throughput proteomics, transcriptomics, and probabilistic graphical models. 

Proteomics has recently been used in the context of advanced melanoma treated with immunotherapy. Garg et al. used a multi-omics approach to characterize samples from long-term and short-term responders to PD-1 immunotherapy. RNA-seq and proteomics pointed out the relevance of the inflammatory response [29]. Harel et al. established a relation between mitochondrial metabolism and the response to tumor-infiltrating lymphocytes or anti-PD-1 antibodies in 116 patients [30]. These investigators recently reported that the concentration of proteins related to immune processes was higher in some metastatic sites such as the lung or the skin, as compared with the brain, lymph nodes or small bowel [31].

In the present study, we used LC-MS/MS proteomics and RNA-seq to characterize 32 samples from patients treated with pembrolizumab or nivolumab. We found that proteins related to protein processing in the ER seem to play a role in the response to anti-PD-1 therapy. This functional node contained relevant proteins that have been related to the immune status of several tumor types. For instance, PDIA6 promotes immune escape in pancreatic cancer through the deubiquitinitation of PD-L1 [32]. CANX (calnexin) has been associated in melanoma with an enhancement in the expression of PD-1 on CD4+ and CD8+ T cells, but also with a promotion of tumor growth and an inhibition of T cell infiltration [33]. PDI proteins are involved in protein folding and antigen presentation machinery (APM). The role of the APM in response to anti-PD-1 immune checkpoint inhibitors in melanoma has been previously described. Thompson et al. defined a signature of eight genes involved in APM (including PDIA3) which predicted responses to immune checkpoint inhibitors in melanoma [34].

The immune response node also had a higher activity in responders than non-responders. The relevance of inflammation in the response to anti-PD-1 inhibitors has been previously reported by Garg et al. [29]. Gide et al. also designed an immune signature containing several immune and inflammatory genes (chemokines and cytokines) to predict responses to single-agent anti-PD-1 therapy and anti-PD-1/anti-CTLA-4 combinations [35]. The immune node in our study contained genes previously associated with a response to immunotherapy or a clinical outcome in melanoma. For instance, CD80 is activated by CTLA-4, and the transfection of human tumor cells with CD80 prevents PD-L1-mediated immunosuppression by tumor cells and restores T cell function [36]. AIM2 is induced by interferon gamma [37] and regulates the stability of regulatory T cells [38]. B2M inactivation by mutation is considered a key point in the resistance to checkpoint inhibitors in melanoma [39]. An absence of B2M leads to the degradation of the MHC I heavy chain [40]. Tumor cells with this phenotype do not express any MHC class I molecules on their surface, so they can escape immunosurveillance and display a higher in vivo tumorigenicity, proliferation rate, and migratory and invasive potential [41]. All this information points to a higher T cell activity in responders than in non-responders and a higher activity of antigen presenting machinery, in line with the results obtained in the node of proteins involved in processing in the ER. Proteins related to protein processing in the endoplasmic reticulum seem to play a role in the response to PD-1 inhibitors as well as immune response, and specifically inflammatory response. This is contrasting with previous studies that established that PDI inhibition promotes the viability of healthy T cells [42]. In addition, a study focused on the characterization of immune cell populations and the response to immunotherapy in melanoma could confirm the results suggested by the immune and inflammatory response node.

Weber et al. used artificial intelligence to build a prognostic predictor based on MS peaks detected in the serum of patients with metastatic melanoma receiving anti-PD-1 therapy [43]. The predictor was validated using an independent cohort [44]. Babacic et al. also studied plasma samples from patients before and during immunotherapy and showed that responders had an increase in proteins related to T cell, neutrophil, inflammatory response, adhesion and immune suppression, and also suggested several proteins that could serve as predictive biomarkers [45]. A two-protein-based predictor was defined in our study. AMBP (alpha-1-microglobulin/bikunin precursor) is the origin of two different proteins: a macroglobulin, which may be involved in the inflammatory response, and bikunin, a urinary trypsin inhibitor. A reduced level of Alpha-1 microglobulin has been previously associated with poor prognosis in renal cell carcinoma [46] and oral squamous cell carcinoma [47]. Alpha-1 microglobulin upregulation in the skin prevents oxidative damage [48]. The second protein in our predictor, PSMD5, is proteasome protein. Harel et al. also highlighted the relevance of proteasome in response to immunotherapy [30], and the role of immuneproteasome related to the response to immunotherapy has also been previously suggested [49].

Our study has some limitations. Validation through an independent cohort of patients with advanced melanoma treated with PD-1 inhibitors is necessary. In addition, the effect of combinatory immunotherapy based on PD-1 and CLTA-4 inhibitors should be taken into account in future studies. 

## 5. Conclusions

To summarize, using high-throughput proteomics, RNA-seq and a multi-omics analysis based on probabilistic graphical models, the molecular landscape of melanoma samples treated with PD-1 inhibitors was characterized. Proteins related to protein processing in the reticulum endoplasmic as well as the immune and inflammatory response processes seem to play a role in the response to PD-1. These biological processes should be further investigated. In addition, a two-protein-based predictor was also defined. The molecular characterization of the mechanisms involved in the response and resistance to immunotherapy in melanoma paves the way to finding therapeutic alternatives for those patients who will not respond to immunotherapy.

## Figures and Tables

**Table 1:** Clinical characteristics of 52 patients with advanced melanoma treated with anti-PD-1 inhibitors.

	Number of Patients (%)
Number of patients	52 (100%)
Age at diagnosis (median and range)	66 (33–88)
Age at diagnosis (mean)	64
Gender	
Male	35 (67.3%)
Female	17 (32.6%)
BRAF mutation	
Positive	15 (28.8%)
Negative	29 (55.8%)
Unknown	8 (15.4%)
Anti-PD-1 treatment	
Pembrolizumab	27 (52%)
Nivolumab	25 (48%)
Best response to anti-PD-1	
CR	11 (21.2%)
PR	13 (25%)
PD	10 (19.2%)
SS	13 (25%)
Non-evaluable	5 (9.6%)
Toxicity to anti-PD-1 treatment	
Yes	10 (19.3%)
No	30 (57.7%)
Unknown	12 (23%)

**Table 2:** Two proteins related to overall survival (*p* < 0.01). HR: hazard ratio.

Protein ID	Gene ID	HR	*p*-Value
P02760	AMBP	1.49	0.0074
Q16401	PSMD5	0.76	0.0077

## Data Availability

The mass spectrometry proteomics data have been deposited to the ProteomeXchange Consortium via the PRIDE partner repository with the dataset identifier PXD038155 and RNA-seq sequencing raw data files are available in Annotare (https://www.ebi.ac.uk/fg/annotare/ (accessed on 30 August 2023)) under the code accession E-MTAB-11729.

## Supplementary Materials

The following supporting information can be downloaded at https://www.mdpi.com/article/10.3390/cancers15174407/s1, Table S1: Clinical data of the 52 patients of the GEM cohort. Table S2: Proteins and genes contained in each functional node of the probabilistic graphical model network. Figure S1: Expression of the main proteins in the node of immune and inflammatory response. CR = complete response; PR = partial response; PD = progressive disease; SD = stable disease.
