# Supplementary figures and images for: Multi-omics Characterization of Response to PD-1 Inhibitors in Advanced Melanoma
Source: Cancers (Basel). 2023 Sep 3;15(17):4407. doi: 10.3390/cancers15174407 (PMC10486782; doi:10.3390/cancers15174407)

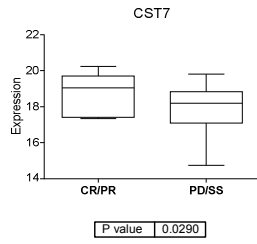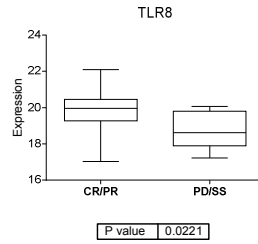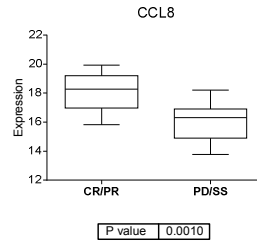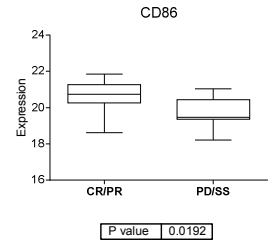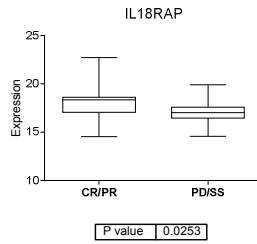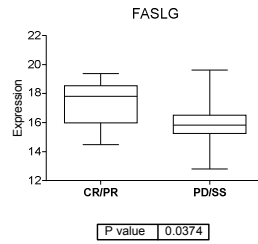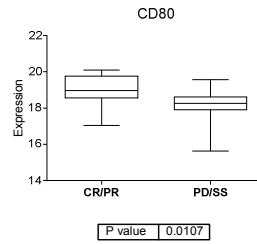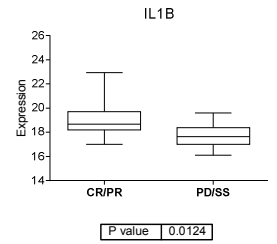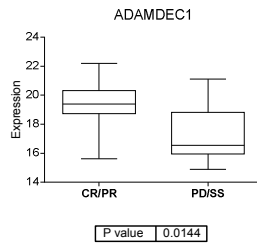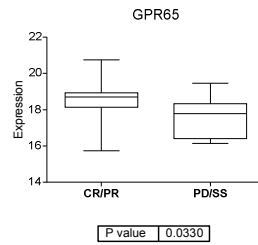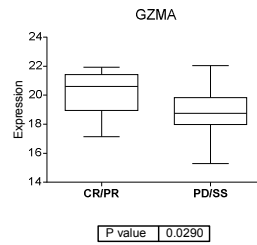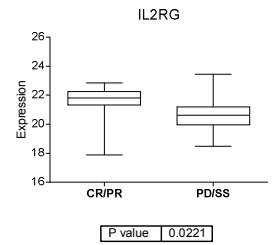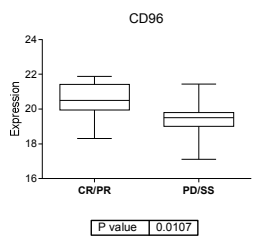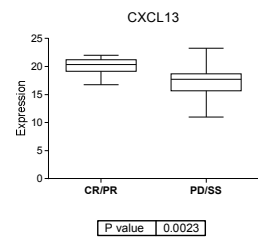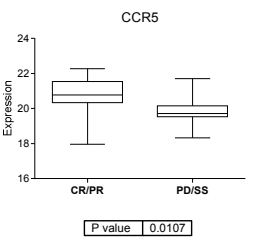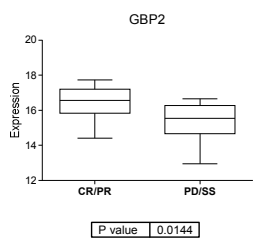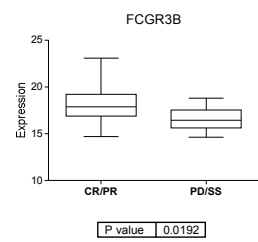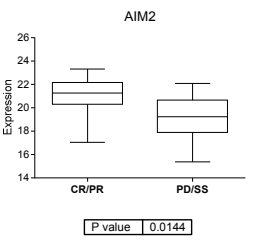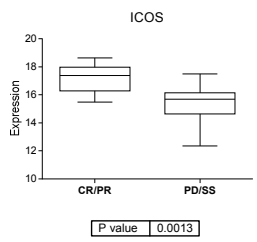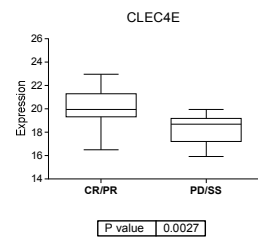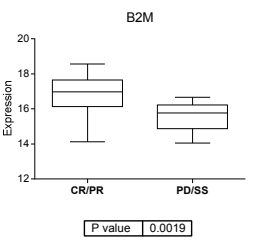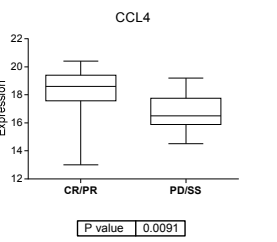

Supplement: Supplementary file 1 [file cancers-15-04407-s001.zip › supplementary Figure S1.pdf]
